# Toward precision medicine in COPD: phenotypes, endotypes, biomarkers, and treatable traits

**DOI:** 10.1186/s12931-025-03356-w

**Published:** 2025-09-29

**Authors:** Cong Xie, Kepeng Wang, Kai Yang, Yuanyuan Zhong, Aman Gul, Weihang Luo, Maimaititusun Yalikun, Jiemin He, Wenjing Chen, Weifang Xu, Jingcheng Dong

**Affiliations:** 1 Institutes of Integrative Medicine, Fudan University, Shanghai, 200032 China; 2 Department of Integrative Medicine, Huashan Hospital Affiliated to Fudan University, Shanghai, 200040 China; 3 School of Clinical Medicine (Yuquan Hospital), Tsinghua University, Beijing, 100040 China; 4 Shenzhen Hospital (Futian) of Guangzhou University of Chinese Medicine, Shenzhen, 518034 China; 5 Department of Integrated Traditional Chinese & Western Medicine, The Second Xiangya Hospital, Central South University, Changsha, 410011 China; 6 Department of Respiratory Medicine, Uyghur Medicines Hospital of Xinjiang Uyghur Autonomous Region, Urumqi, 830049 China

**Keywords:** Chronic obstructive pulmonary disease (COPD), Precision medicine, Phenotype, Endotype, Biomarker, Treatable traits, Targeted therapies

## Abstract

Chronic obstructive pulmonary disease (COPD) is a heterogeneous disorder characterized by diverse clinical manifestations, pathophysiological mechanisms, and therapeutic responses. This review explores the evolving landscape of precision medicine in COPD management, with particular emphasis on optimizing patient care through the integration of phenotypes, endotypes, biomarkers, and treatable traits. Phenotypic classification based on observable clinical and radiographic features has facilitated the identification of distinct subgroups such as “emphysema-dominant” or “frequent-exacerbator” subtypes. Emerging research, however, increasingly emphasizes endotypes—disease subcategories defined by unique biological mechanisms including neutrophilic inflammation, eosinophilic airway involvement, or α_1_ antitrypsin deficiency—which may demonstrate superior predictive value for therapeutic responses. Biomarkers encompassing blood eosinophil counts, serum C-reactive protein, and sputum transcriptomics are progressively being implemented for patient stratification and guidance of targeted therapies, including inhaled corticosteroids or biologics. Furthermore, the “treatable traits” framework enhances personalized management by addressing modifiable factors beyond airflow limitation, such as comorbidities, psychosocial determinants, and exacerbation triggers. Despite these advancements, persistent challenges remain in biomarker validation, standardization of phenotypic definitions, and clinical translation of research findings. Future directions involve early detection of pre-COPD states and treatable traits, integration of multi-omics data, machine learning-driven dynamic phenotyping, and pragmatic clinical trials evaluating precision-guided interventions. By aligning pathobiological mechanisms with targeted therapies, precision medicine holds promise for transforming COPD care from reactive management to proactive, individualized therapeutic paradigms.

## Introduction

Chronic obstructive pulmonary disease (COPD) stands as one of the globe’s predominant respiratory health challenges, characterized by significant disability and mortality rates [1]. This condition is driven by chronic inflammation, typically associated with prolonged tobacco use or exposure to noxious substances [2]. However, emerging perspectives recognize COPD not as a singular disease entity but rather as a complex heterogenous syndrome manifesting marked interindividual variability in clinical presentations, lung function parameters, and pathophysiological mechanisms, thereby necessitating its conceptualization as an umbrella term encompassing multiple clinical subtypes [3]. The pathogenesis of COPD extends beyond smoking-related damage, being shaped by genetic susceptibility and lifetime exposure patterns (commonly termed the “exposome”). Early-life determinants (including premature birth, childhood respiratory infections, or asthma), chronic air pollution exposure, occupational hazards, and socioeconomic factors collectively contribute to heterogeneous lung development trajectories and injury patterns [4]. These divergent pathogenic pathways culminate in distinct disease phenotypes. For instance, regional epidemiological patterns reveal heightened COPD risk among women exposed to indoor biomass fuels despite non-smoking status [5, 6]. Such complexity renders universal management strategies suboptimal, given substantial interpatient variability in therapeutic responses and clinical outcomes.

The recent paradigm shift toward precision medicine has catalyzed efforts to tailor interventions according to individual disease characteristics, transitioning from symptom-based classification to a more detailed, mechanism-oriented framework. Key concepts for achieving this goal include phenotypes (observable clinical manifestations), endotypes (molecular or pathophysiological subtypes), and treatable traits (detectable and targetable disease features within individuals) [7, 8]. These distinctions are increasingly supported by biomarker discoveries derived from multi-omics technologies (e.g., genomics, proteomics), which hold promise for predicting disease trajectories and therapeutic responsiveness. Leveraging these conceptual lenses, clinicians and researchers are redefining COPD into mechanistically distinct subgroups with identifiable therapeutic targets. This review synthesizes recent advancements in COPD phenotyping, endotype characterization, and biomarker identification, explores molecular underpinnings of disease heterogeneity, and delineates how the treatable traits framework may operationalize precision medicine in COPD management.

## The heterogeneity of COPD

COPD is a highly heterogeneous respiratory disorder with pathological features varying in multiple aspects, including the degree of airway remodeling, inflammatory response, airflow distribution, and the extent of alveolar emphysema, etc [3]. These pathological changes lead to progressively worsening airflow limitation in patients, resulting in increased breathing difficulty, disability, and even death [9]. Additionally, lung function in COPD patients also exhibits heterogeneity, with the rate of lung function decline closely related to the initial percentage of forced expiratory volume in one second (FEV_1_) relative to the predicted value and the Global Initiative for Chronic Obstructive Lung Disease (GOLD) grade [10]. This heterogeneity complicates the clinical features of COPD, with varying proportions of clinical characteristics occurring among different patients [11], indicating that COPD is not a single, simple disease but rather a collection of diverse conditions. Therefore, individualized treatment and management strategies are necessary. With the accumulation of evidence-based medical research, the definition of COPD has become increasingly clear and tends towards scientific precision [12]. The definition of the disease in the GOLD guidelines has also gradually evolved: in 2001, the irreversibility of airflow limitation was emphasized; [13] in 2006, COPD was defined as a disease characterized by “chronic airflow limitation” and “a series of pathological changes in the lungs,” with significant extrapulmonary manifestations and important comorbidities that may exacerbate the severity of the patient’s condition, highlighting that COPD can be prevented and treated; [14, 15] GOLD 2011 considered COPD to be characterized by “progressive persistent airflow limitation,” associated with enhanced chronic inflammation of the airways and lungs due to toxic particles or gases [15]. Notably, the 2017 revision expanded the COPD definition to incorporate “persistent respiratory symptoms” alongside sustained airflow limitation, while substituting “chronic inflammatory response” with “airway and/or alveolar abnormalities caused by toxic particles or gases” to emphasis structural damage; meanwhile, in the pathophysiological section, it was stated that the occurrence and development of COPD change over time, highlighting host factor contributing to disease pathogenesis [16]. And then, in 2023, the GOLD guidelines explicitly defined the “heterogeneity” of COPD and its clinical symptoms, pathological alterations, and physiological characteristics [17]. 

The 2023 GOLD report acknowledges the etiologic heterogeneity of COPD and introduces a novel classification methodology termed “**etiotypes**,” which categorizes COPD based on predominant risk factors [8, 17]. The report classifies COPD into seven distinct etiologic subtypes: (1) genetically determined COPD (COPD-G), (2) COPD due to abnormal lung development (COPD-D; e.g., caused by early-life events), (3) cigarette smoking COPD (COPD-C), (4) biomass and pollution exposure COPD (COPD-P), (5) COPD due to infections (COPD-I; e.g., post-tuberculosis), (6) COPD with asthma (COPD-A), and (7) COPD of unknown cause (COPD-U) [8, 18]. This taxonomic framework underscores the multifactorial nature of COPD pathogenesis across global populations. For example, non-smoking COPD (from biomass or repeated childhood infections) is highly prevalent in low- and middle-income countries [19]. Distinct clinical phenotypes may characterize specific etiologic subtypes – for instance, biomass-associated COPD frequently manifests greater airway fibrosis with less emphysematous destruction compared to tobacco-related disease [19]. However, clinical complexity arises from frequent etiologic subtype overlap (e.g., tobacco users with concomitant early-life developmental lung impairment), and the therapeutic implications of such multimorbidity remain undefined. Nonetheless, recognition of etiologic and geographic disparities proves critical for developing precision medicine strategies at a global scale. This paradigm reinforces the imperative for implementing clean energy cooking fuel initiatives (targeting biomass exposure mitigation) and early-life health preservation programs (optimizing pulmonary development) as integral components of comprehensive COPD prevention and management frameworks. Beyond energy transition and early-life lung development, optimizing asthma control and preventing respiratory infections, for example by vaccinating against influenza, pneumococcus, and RSV, are also pivotal levers across the life-course to reduce future COPD risk and acute exacerbations [20]. 

Besides causal factors, COPD heterogeneity is evident in its pathophysiology and clinical course. Some patients experience frequent acute exacerbations while others remain stable; [21] some have a steady decline in lung function whereas others progress more slowly [22, 23]. Comorbidities also vary among individuals. COPD commonly overlaps with cardiovascular disease, osteoporosis, lung cancer, depression, and others, each of which can influence symptoms and outcomes [24]. All these layers of heterogeneity mean that treatments effective in one subgroup may be less effective in another. Indeed, heterogeneity in clinical characteristics and underlying biology is a major determinant of therapeutic response. This reality has propelled the identification of clinically meaningful phenotypes and endotypes within “COPDs,” moving away from a one-size-fits-all approach. In summary, appreciating the heterogeneity of COPD across different populations, risk factors, and biologic profiles represents the first step towards precision medicine that delivers the right interventions to the right patient.

## Clinical phenotypes of COPD

**Fig. 1:**
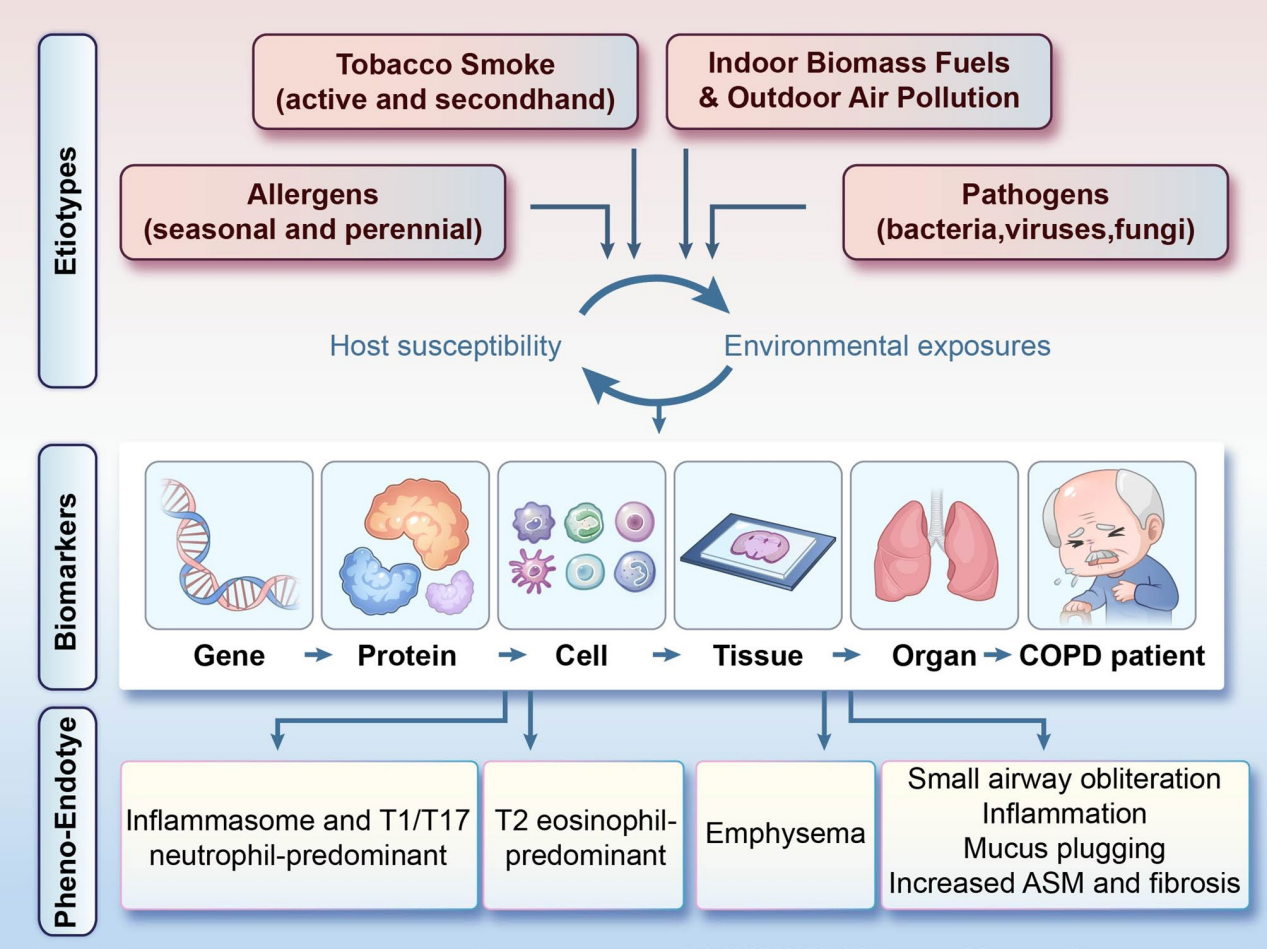
Multi-scale heterogeneity in COPD. COPD arises through dynamic interactions across genetic, cellular, and systemic levels, driven by individual variations in host-environment responses that shape distinct clinical phenotypes and endotypes. *ASM* Airway smooth muscle

Moving from causes to consequences, COPD heterogeneity becomes apparent in diverse clinical phenotypes, which are further subdivided by observable clinical features and prognostic differences. The clinical phenotypes of COPD refer to one or several disease characteristics that can reflect the differences among COPD patients, and are associated with clinical outcomes (e.g., symptoms, exacerbations, treatment responses, disease progression rates, or mortality) [25]. As research advances, the understanding of COPD etiology has expanded from “exposure to harmful gases/particles” to “multiple etiological types.” [13, 17] The 2023 GOLD guidelines introduced a new category based on etiology, extending to non-smoking-related types [17]. This new classification provides a foundation for identifying distinct pathological and physiological mechanisms in COPD, facilitating advancements in prevention, therapeutic innovation, and precision medicine development (Fig. 1).

It is important to clarify the conceptual distinction between phenotypes and endotypes to avoid confusion. A phenotype refers to a collection of observable clinical characteristics such as symptoms, exacerbation frequency, lung function trajectories, or imaging patterns that can be readily identified in clinical practice. In contrast, an endotype represents the underlying biological or molecular mechanisms that give rise to these observable traits. Notably, a single phenotype may correspond to multiple distinct endotypes. For example, the frequent exacerbator phenotype may result from an eosinophilic inflammation driven endotype or from an infection dominant endotype, each with distinct therapeutic implications. This layered framework highlights why phenotypic classification is clinically valuable but ultimately requires integration with mechanistic endotypes for precision management.

### Chronic bronchitis and emphysema phenotypes of COPD

The first edition of the GOLD guidelines, published in 2001, did not differentiate between emphysema and chronic bronchitis [13]. However, the 2023 update has reinstated the emphasis on these concepts, returning to the fundamental nature of COPD [17]. The emphysematous phenotype (historically labeled “pink puffer,” type A) predominantly affects elderly, underweight individuals, presenting with pronounced symptoms yet milder inflammation (absence of cough or sputum production), no cyanosis, and arterial blood gas analyses showing a slight decrease in PaO_2_ and normal or reduced PaCO_2_ levels. These patients exhibit extensive alveolar destruction on computed tomography (CT) scans, leading to diminished diffusion capacity and pulmonary hyperinflation; Clinically, they manifest with significant dyspnea, accompanied by weight loss and muscle wasting. The severity of emphysema correlates with exercise limitation and can predict the benefits of certain interventions, such as upper lobe-predominant lung volume reduction surgery. In contrast, the chronic bronchitis phenotype (formerly known as “blue bloater,” type B), more common in younger, obese individuals, is characterized by pronounced symptoms and inflammation, yet less severe emphysema. It features significant airway obstruction, cyanosis, markedly reduced PaO_2_, and elevated PaCO_2_ levels, frequently associated with respiratory failure. Studies indicate that patients with this phenotype of COPD experience more exacerbated symptoms [26], poorer lung function, lower Quality of Life, and a 1.3-fold increased risk of mortality [27, 28]. Given their higher risk of acute exacerbations, it is recommended that patients with chronic bronchitis phenotype COPD undergo long-term maintenance therapy with a combination regimen including inhaled corticosteroids (ICS), such as ICS/long-acting β-agonist (LABA) or triple therapy with ICS/LABA/long-acting muscarinic antagonist (LAMA). Support for this recommendation includes trial evidence: the SUMMIT study demonstrated that ICS/LABA (fluticasone furoate/vilanterol) significantly reduced the rate of moderate‑to‑severe exacerbations in moderate COPD patients at high cardiovascular risk (*P* < 0.025) [29], and the TRIBUTE trial showed that single‑inhaler extrafine triple therapy (beclometasone dipropionate [BDP→ICS]/formoterol fumarate [FF→LABA]/glycopyrronium [G→LAMA]) reduced exacerbation rates by approximately 15% compared to LABA/LAMA therapy [30]. This phenotype is associated with neutrophilic airway inflammation and may respond favorably to specific treatments, such as mucolytics and phosphodiesterase-4 (PDE4) inhibitors [31]. 

### COPD with idiopathic pulmonary fibrosis (IPF)

Interstitial lung abnormalities (ILA) refer to incidental findings of pulmonary interstitial anomalies in patients without known interstitial lung diseases (ILD) [32]. Studies have shown that the prevalence of ILA ranges from 4 to 9% among individuals over the age of 60 [33]. In the COPDGene cohort study, approximately 8% of COPD patients were found to have ILA, half of whom met the criteria for suspected ILD based on CT evidence of definitive fibrosis, forced vital capacity (FVC) less than 80% of the predicted value, or carbon monoxide diffusing capacity (DL_CO_) less than 70% of the predicted value [34]. These patients with suspected ILD typically exhibit more pronounced respiratory symptoms and have higher mortality rates. Fibrotic ILA, characterized by traction bronchiectasis, architectural distortion, and honeycombing on imaging, is more likely to progress and is associated with poor prognosis, especially in patients with concurrent emphysema [35]. Consequently, COPD patients with imaging findings suggestive of pulmonary fibrosis warrant comprehensive clinical evaluation and risk stratification, coupled with ongoing monitoring to inform personalized treatment strategies aimed at mitigating poor prognoses.

### Non-smoking COPD

Non-smoking-related COPD accounts for 20–50% of cases across both developed and developing countries [5], with a higher proportion in low-income regions largely attributable to biomass exposure. The Burden of Obstructive Lung Disease (BOLD) programme [36] further highlights non-smoking risk factors, such as low educational attainment, occupational exposures, low body mass index, and a history of tuberculosis, although relative risks vary by setting [4]. Beyond association, occupational exposures represent an important and preventable cause of COPD: the ATS/ERS statement estimates a population-attributable fraction of ~ 14% for COPD (and ~ 13% for chronic bronchitis), underscoring causal impact at the population level [37]. At the job level, analyses of lifetime work histories in the UK Biobank identified six occupations with increased COPD risk [38], findings that remained in never-smokers and never-asthmatics and showed exposure response trends, while agent-based modeling in the same cohort implicated pesticides with robust, dose-dependent associations [39]. In most provinces of China, smoking prevalence among males exceeds 50%, whereas female COPD is more strongly influenced by passive smoking, indoor and outdoor air pollution, and occupational particulate exposure [40]. Representative non-smoking COPD subtypes are increasingly recognized, including biomass-related COPD, post-tuberculosis obstructive disease, developmental or dysanapsis-related COPD due to impaired lung growth, occupational dust- or chemical exposure–related COPD, and genetic forms such as α_1_ antitrypsin deficiency (AATD) [19]. Recognition of these subtypes is important, as their risk factors, clinical features, and therapeutic implications may differ substantially from tobacco-related COPD, underscoring the need for tailored prevention and management strategies.

### Acute exacerbation of COPD (AECOPD)

AECOPD is defined as a critical clinical phase in COPD patients characterized by worsening of dyspnea, cough, and sputum production within 14 days, potentially accompanied by tachypnea and tachycardia. These episodes are typically triggered by enhanced local and systemic inflammatory responses associated with respiratory infections, air pollution, or other factors causing airway damage [17]. As a hazardous stage and pivotal event in COPD progression, the severity and frequency of exacerbations profoundly impact patients’ Quality of Life and survival duration. Approximately 25% of COPD patients experience accelerated lung function decline attributable to acute exacerbations [41], while those with impaired pulmonary function face heightened risks of future exacerbations [42]. Disease severity correlates positively with exacerbation frequency, leading to the identification of a frequent-exacerbation phenotype defined as ≥ 2 annual events [43]. Key risk factors for frequent exacerbations include persistent blood eosinophilia, poor baseline health status, gastroesophageal reflux, lower respiratory tract bacterial colonization, and chronic mucus hypersecretion [44]. Numerous studies have explored predictive methodologies for COPD exacerbation risk, revealing elevated levels of decorin and α-macroglobulin as potential biomarkers for frequent exacerbations [45]. Notably, a quantitative CT-based predictive model developed from the U.S. SPIROMICS cohort demonstrates capability to identify high-risk patients for severe exacerbations [45, 46]. During COPD exacerbations, there is a reduction in lung microbial diversity, with certain bacterial genera (such as *Haemophilus* and *Moraxella*) showing a clear association with the severity, progression, and bronchiectasis of COPD [47]. Consequently, the development of models based on lung microbiota holds promise for better predicting the risk of COPD exacerbations and guiding targeted therapies [47]. 

### Asthma-COPD overlap (ACO)

ACO also constitutes a clinically significant phenotype, characterized by patients exhibiting features of COPD alongside a history of asthma or evidence of allergy, positive bronchodilator response, or eosinophilic inflammation [48]. These individuals manifest dual characteristics of both asthma and COPD, including relatively preserved diffusion capacity and elevated levels of IgE or blood eosinophils. Patients with ACO typically present with persistent airflow obstruction that demonstrates both irreversible components (resembling COPD) and reversible elements (analogous to asthma), rendering diagnosis and therapeutic management particularly challenging. Such individuals frequently experience recurrent acute exacerbations and respond less favorably to conventional therapies designed for either asthma or COPD alone. Furthermore, ACO patients may exhibit heightened sensitivity to environmental triggers such as smoke, cold air, or physical exertion, exacerbating their symptoms. Given the incomplete understanding of the pathophysiological mechanisms underlying this overlapping condition, optimal clinical management remains an evolving area of investigation [49]. Current guidelines recommend triple therapy (ICS/LABA/LAMA) for this population over monotherapy with ICS, LABA/LAMA dual therapy, or ICS/LABA combinations [7]. This clinical entity underscores the diagnostic and nosological challenges posed by the blurred boundaries between asthma and COPD in specific individuals, highlighting the need for phenotype-driven therapeutic approaches.

### Early disease States of COPD

The lung function trajectories of COPD patients exhibit significant heterogeneity [10, 50]. Given our current inability to reverse pulmonary damage, defining early-stage COPD becomes crucial for mitigating disease progression [51]. Although established criteria exist for identifying mild disease manifestations, a specific consensus definition for the early phase of COPD remains elusive [51]. Early COPD should ideally be defined by the initial pathophysiological changes that ultimately lead to disease development, though such precise characterization remains currently unattainable [51]. Since 2022, the GOLD guidelines have proposed several concepts for early disease states of COPD, including pre-COPD, early-onset COPD in young adults, and Preserved Ratio Impaired Spirometry (PRISm) [52]. Identifying individuals within these definitions and paying attention to their clinical characteristics and mechanistic studies may deepen our understanding of COPD and aid in improving treatment strategies.

Pre-COPD is considered a potential precursor stage of the disease, defined as having abnormal spirometric measurements or significant emphysema shown on chest CT scans without airflow obstruction. A population-based study employing CT or microCT quantification found that compared to controls, individuals with pre-COPD and those across all GOLD grades had a significant reduction in the number of transitional bronchioles (TrB)/ml and terminal bronchioles (TB)/ml.^53^ Additionally, TrB and TB had fewer alveolar attachment points, with no differences observed between pre-COPD and established COPD groups [53]. Histopathological analysis revealed that, compared to controls, both pre-COPD and all GOLD grades exhibited an increase in mean alveolar intercept and a decrease in alveolar surface density [53]. These findings suggest that pre-COPD patients with emphysematous changes already exhibit pathological features mirroring established COPD, including reduced small airway numbers and airway remodeling, despite preserved airflow metrics. To explore clinical biomarkers for pre-COPD, the Tasmanian Longitudinal Health Study (TAHS) longitudinally monitored lung function in a 45-year-old cohort over 8 years, identifying suboptimal pulmonary performance without overt airflow obstruction as a potential pre-COPD marker [54]. Utilizing the 10th percentile of the FEV_1_/FVC ratio before inhaling bronchodilators (*z* score < −1.264) as a threshold, the risk of developing COPD could be accurately predicted with high sensitivity (88%) and specificity (87%).^54^ Participants below this threshold had a 36-fold increased risk of developing COPD during the 8-year follow-up period, especially among males who were current smokers with comorbid asthma [54]. Therefore, simple spirometric measurements can identify individuals at high risk of COPD in community or primary care settings, offering an opportunity to slow or even prevent disease progression.

Early-onset COPD in young adults is defined as occurring in individuals aged 20–50 years who never achieved normal peak lung function in early adulthood or experienced an accelerated decline in lung function. The future development and progression of COPD in this group have attracted attention. Investigations have operationalized COPD-susceptible states as chronic bronchitis, PRISm, or early airflow limitation (FEV_1_/FVC ≥ 0.70 but < lower limit of normal). Longitudinal analyses from two population cohorts revealed divergent outcomes: [55] the Copenhagen General Population Study (CGPS) demonstrated 28% COPD incidence (13% GOLD 2–4) among susceptible individuals versus 8% (1% GOLD 2–4) in non-susceptible controls over 10 years. The Copenhagen City Heart Study (CCHS) extended these observations over 25 years, documenting 22% versus 13% COPD incidence and 20% versus 8% GOLD 2–4 progression in susceptible versus non-susceptible groups respectively. Multivariable-adjusted analyses revealed substantial risk elevations: odds ratios (OR) of 3.42 (95% *CI*: 2.78–4.21) for COPD and 10.1 (95% *CI*: 6.77–15.2) for GOLD 2–4 at 10 years, declining to 1.54 (95% *CI*: 1.23–1.93) and 2.12 (95% *CI*: 1.64–2.73) respectively at 25 years. These findings substantiate the existence of a detectable preclinical phase, emphasizing the imperative for early detection and preventive strategies [55]. 

The concept of PRISm has garnered significant attention since its introduction, recognized as a transitional state toward COPD and a high-risk subtype prone to COPD development or acute exacerbations [56]. Mechanistically, PRISm arises from several non-mutually exclusive pathways: some patients exhibit small-airway dysfunction with gas trapping indicative of early obstructive disease, others demonstrate obesity-related restrictive ventilatory mechanics, and still others reflect impaired lung development or dysanapsis, in which airway–parenchyma mismatch reduces ventilatory reserve despite a preserved FEV_1_/FVC ratio [56, 57]. A retrospective South Korean study utilizing data from the 2007–2012 Korean National Health and Nutrition Examination Survey investigated the clinical characteristics and healthcare utilization patterns among smokers with PRISm [58], revealing comparable healthcare utilization rates between PRISm and COPD patients. Factors associated with increased medical resource use in PRISm included advanced age, female sex, heavy smoking, low body mass index (BMI), lower socioeconomic status, limited educational attainment, wheezing, and reduced FEV_1_ and FVC% predicted.^58^ Furthermore, an analysis of the Canadian Cohort Obstructive Lung Disease (CanCOLD) data demonstrated that both PRISm and COPD patients exhibited significantly higher prevalence of cardiovascular comorbidities, particularly ischemic heart disease and heart failure, compared to individuals with normal spirometry [59]. The substantial healthcare burden associated with PRISm highlights the critical need for early intervention strategies in this population.

Prognostically, PRISm is clinically important because approximately one-third of patients progress to overt COPD, while others remain stable or even revert to normal spirometry. In addition to pulmonary outcomes, PRISm is strongly associated with increased cardiometabolic comorbidities and higher all-cause mortality. Imaging adds prognostic granularity: parametric response mapping (PRM) can help distinguish airway-dominant versus emphysema-dominant trajectories, providing early risk stratification. Notably, PRISm demonstrates higher prevalence among males and active smokers—groups already identified as high-risk populations for COPD [60, 61]. Smoking not only accelerates the decline in FEV_1_ and FVC but also elevates the likelihood of PRISm progression to overt COPD [56]. Longitudinal evidence supports that about one-third of PRISm cases ultimately progress to COPD diagnosis [60, 62]. Disease trajectories vary significantly across PRISm subtypes, with mild cases exhibiting greater potential for lung function normalization, while severe PRISm tends to evolve into advanced COPD stages [63]. These observations emphasize the importance of risk stratification and tailored management approaches based on disease severity.

In summary, the aforementioned phenotypes illustrate the diverse manifestations of COPD across individuals. Importantly, these phenotypes are not mutually exclusive. For instance, an individual may present as an emphysema-dominant frequent exacerbator with concurrent ACO features. Nevertheless, phenotypic classification holds significant clinical implications, enabling clinicians to tailor therapeutic strategies accordingly. For example, patients with eosinophilic ACO phenotypes may respond favorably to inhaled corticosteroid therapy, whereas those with pure emphysema lacking eosinophilic inflammation may not [64]. Clinical guidelines have indeed incorporated phenotypic characteristics—often termed treatable traits—into therapeutic recommendations [7]. The Spanish COPD guidelines (GesEPOC) represent an early adopter of phenotype-directed management, offering distinct therapeutic algorithms for ACO, chronic bronchitis, or frequent exacerbators [65]. More recently, the GOLD strategy has shifted from rigid spirometric classification to an ABCD assessment incorporating symptom burden and exacerbation history—a tacit acknowledgment of phenotypic variability in symptom profiles and risk stratification [66]. The integration of blood eosinophil counts to identify corticosteroid-responsive patients further marks a stride toward precision medicine [64]. Despite their utility, phenotypes inherently possess limitations. They are grounded in clinical manifestations that may arise from diverse underlying biological mechanisms. The “frequent exacerbator” phenotype, for example, may encompass heterogeneous endotypes—some individuals experience exacerbations driven by eosinophilic airway inflammation (steroid-responsive), while others suffer recurrent infections due to impaired host defense or bronchiectasis (potentially more amenable to antibiotic strategies). Phenotypic classification alone fails to fully capture such pathophysiological nuances, thereby necessitating the concept of endotypes—molecular or cellular drivers of disease that operate within or across phenotypic categories.

## Molecular endotypes of COPD

An endotype is defined as a subtype of a disease condition characterized by a specific pathobiological mechanism or molecular profile, which may or may not correspond directly to a visible clinical phenotype [8]. Endotypes in COPD seek to answer why a given patient has the features they have – for example, is a patient’s airflow limitation driven predominantly by eosinophilic inflammation, neutrophilic inflammation, autoimmunity, protease-mediated tissue destruction, or other mechanisms? By identifying endotypes, clinicians can target the underlying biology of the disease in each patient, moving closer to true precision medicine. Research into COPD endotypes is rapidly evolving, with several specific endotypes having been proposed or identified. Whereas phenotypes represent the outward clinical manifestations of COPD, endotypes provide the mechanistic explanations underlying those manifestations, thereby bridging clinical observation with molecular pathogenesis.

### Neutrophil-predominant (“type 2-low”) endotype

**Fig. 2:**
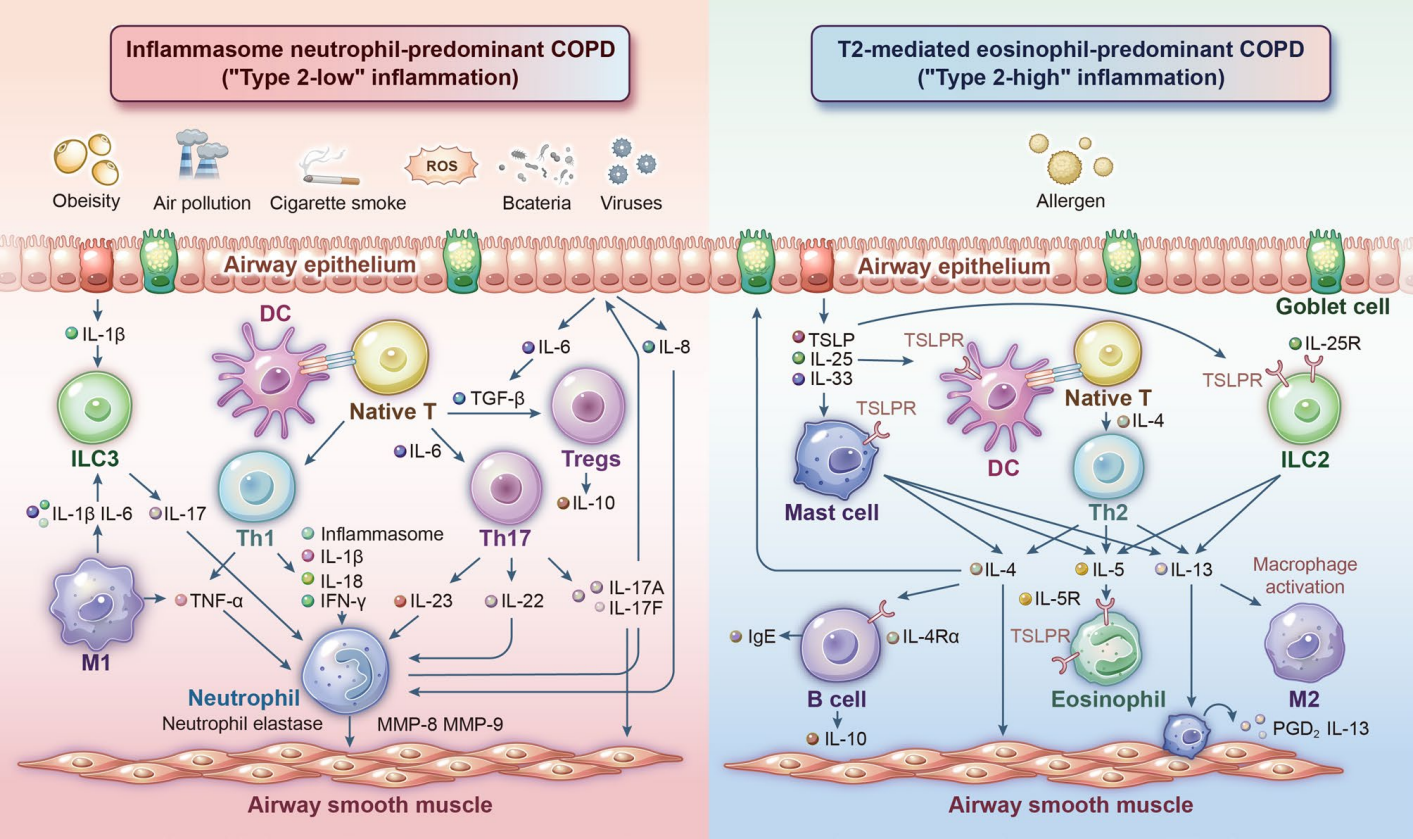
Dichotomous cytokine profiles in COPD endotypes: neutrophil-centric inflammasome pathway (“T2-low” COPD) versus eosinophil-dependent type 2 immunity (“T2-high” COPD), depicting mechanism-specific responses to environmental stimuli. *DC* Dendritic cell, *MMP* Matrix metalloproteinase, *PGD* Prostaglandin D, *TSLP* Thymic stromal lymphopoietin

This endotype is defined by dominant airway neutrophilic inflammation mediated through interleukin (IL)−1, IL-8, and Th17 (type 17 T helper cell)/IL-17 pathways, distinct from type 2 cytokine-driven mechanisms. It aligns with the classical smoking-associated chronic bronchitis phenotype, clinically characterized by chronic cough, sputum hypersecretion, bacterial colonization, and inflammation resistant to corticosteroid therapy. Biologically, these patients demonstrate elevated IL-17 levels and neutrophil-specific chemotactic factors, underpinned by a well-defined molecular cascade (Fig. 2). Environmental insults, including cigarette smoke, biomass exposure, oxidative stress, and microbial pathogens, activate neutrophilic inflammation via nuclear factor-κB (NF-κB) and p38 mitogen-activated protein kinase (MAPK) signaling in airway epithelial cells [67]. This triggers the release of damage-associated molecular patterns (DAMPs; e.g., IL-33, thymic stromal lymphopoietin [TSLP]) and neutrophil chemoattractants such as CXCL1 and CXCL8 (signaling through CXCR2 receptors) and leukotriene B4 (LTB4; via BLT1 receptors) [67]. CCL2 recruits monocytes that differentiate into macrophages, which produce IL-23 to attract Th17 and group 3 innate lymphoid cells (ILC3 cells), thereby amplifying IL-17 production [68]. IL-17 further stimulates epithelial secretion of IL-6 and CXCL8 [68]. TNF-α and GM-CSF sustain neutrophilic persistence in airways, promoting neutrophil elastase (a potent mucin secretagogue) and matrix metalloproteinase (MMP)−9 release [68, 69]. Neutrophil-derived oxidative stress exacerbates inflammation and induces corticosteroid resistance [68]. Concurrently, TGF-β and connective tissue growth factor (CTGF) released from epithelial and immune cells drive fibroblast proliferation and peribronchial fibrosis [69]. 

Clinically, this endotype correlates with accelerated lung function decline and recurrent infectious exacerbations. Given the limited efficacy of ICS—and potential pneumonia risk—alternative therapies targeting neutrophilic pathways are under investigation. Anti-IL-17 biologics (e.g., secukinumab) show preliminary potential in attenuating airway remodeling in neutrophilic-dominant patients, though clinical validation remains pending [70]. Macrolides, via immunomodulatory and antimicrobial effects, may benefit patients with chronic airway infection, while PDE4 inhibitors (e.g., roflumilast) target neutrophilic inflammation in chronic bronchitis [71]. 

An infectious sub-endotype, marked by acute/chronic airway infections, may exist within this spectrum. Impaired barrier function predisposes to microbial dysbiosis and colonization, with pollutants driving ILC2-to-ILC1 polarization, amplifying type 1 inflammatory cascades [72]. In COPD, Proteobacteria dominance (e.g., *Haemophilus influenzae*, *Pseudomonas*) correlates with elevated γ-Proteobacteria: Firmicutes (γP: F) ratios [73–76]. These pathogens perpetuate inflammation via pathogen-associated molecular pattern (PAMP) activation, with *H. influenzae* abundance directly linked to disease severity [77, 78]. Therapeutic strategies focus on infection control through prophylactic antibiotics (e.g., long-term macrolides), mucociliary clearance techniques, inhaled antimicrobials, and emerging microbiome-modulating therapies.

### Eosinophil-predominant (“type 2-high”) endotype

Initially, COPD-related inflammation was considered to be solely driven by type 1 immune responses, involving CD4^+^ Th1 cells, CD8^+^ cytotoxic T (Tc1) cells, macrophages, and neutrophils [69]. However, emerging research has highlighted a distinct inflammatory subgroup characterized by type 2 inflammation, termed the eosinophil-predominant or “type 2-high” COPD endotype [79]. This subgroup accounts for 20–40% of COPD patients and is defined by elevated eosinophil levels in sputum and/or blood, along with activation of type 2 immune pathways—features reminiscent of asthma [79]. These individuals exhibit heightened risks of acute exacerbations (95% *CI*: 1.10–1.63) [44, 80] and hospital readmissions [81]. Clinically, this endotype often overlaps with the ACO phenotype or demonstrates “asthma-like” traits, including increased reversibility to bronchodilators, favorable responses to ICS, elevated fractional exhaled nitric oxide (FeNO), and sputum eosinophilia [82]. Blood eosinophil counts are now utilized as biomarkers to predict ICS therapeutic benefits [83]. 

The type 2 inflammatory milieu in COPD involves multiple cellular players, including Th2 cells, ILC2s, B cells, eosinophils, basophils, mast cells, dendritic cells (DCs), and M2 macrophages [84–86]. Key cytokines include IL-4, IL-13, IL-5, IL-33, IL-25, TSLP, chemokines, and IgE [84, 85]. Th2 cells and ILC2s are central drivers: Th2 cells regulate humoral immunity, amplify adaptive immune responses, and activate innate immune cells, while ILC2s modulate anti-infective defenses and inflammatory homeostasis (Fig. 2). Among cytokines, IL-4, IL-13, and IL-5 exhibit both distinct and overlapping roles [87]. IL-4 promotes Th0-to-Th2 differentiation [87], whereas IL-13 governs goblet cell hyperplasia, mucociliary dysfunction, mucus hypersecretion, airway smooth muscle hypertrophy, fibroblast proliferation, and collagen deposition [87–90]. IL-5 specifically regulates eosinophil differentiation and survival [91]. Synergistically, IL-4 and IL-13 drive M2 macrophage polarization [86], B cell class-switching, IgE production, mast cell degranulation, epithelial barrier dysfunction [92], airway hyperresponsiveness [93], fibrotic remodeling, and emphysema [87, 94, 95]. All three cytokines contribute to eosinophil recruitment into lung tissues [94]. Notably, IL-4 and IL-13 exert broader influences across upstream and downstream pathological cascades, positioning them as core regulators of type 2 inflammation. In contrast, IL-5 appears confined to eosinophil-related mechanisms.

Pathologically, IL-4– and IL-13–mediated processes align with hallmark COPD features such as airway barrier disruption, mucus plug formation, fibrotic remodeling, and emphysema. These changes culminate in airflow limitation, persistent dyspnea, chronic cough, progressive lung function decline, and acute exacerbations. Therapeutically, precision approaches targeting type-2/eosinophilic inflammation have matured. Anti-IL-5 therapy with mepolizumab reduced annualized moderate–severe exacerbations when added to triple therapy in eosinophil-high COPD (MATINEE phase 3), providing confirmatory evidence for IL-5 pathway inhibition in appropriately enriched populations [96]. In contrast, anti-IL-5Rα therapy with benralizumab did not meet primary endpoints in GALATHEA/TERRANOVA overall, although exploratory signals in higher-eosinophil subgroups highlight the importance of biomarker-based selection [97]. Beyond IL-5, IL-4Rα blockade with dupilumab lowered exacerbation rates and improved lung function and symptoms in two independent phase-3 trials (BOREAS, NOTUS) among patients with evidence of type-2 inflammation [98, 99]. Taken together, these data support biomarker-guided use of ICS and biologics in the eosinophil-predominant endotype, with a continuous gradient of ICS benefit (minimal at < 100 cells/µL; greatest at ≥ 300 cells/µL).

### Rare genetic endotypes

Certain rare genetic mutations cause COPD via specific molecular mechanisms. The best-known example is Alpha-1 Antitrypsin (AAT) deficiency, caused by mutations in the *SERPINA1* gene [100]. This endotype involves unchecked neutrophil elastase activity leading to early-onset panacinar emphysema [101]. It represents a proof-of-concept for endotype-specific therapy, as augmentation with purified AAT protein markedly alters the disease course in these patients [102]. Another genetic endotype involves telomerase gene (TERT) mutations or telomere biology disorders, which cause premature cellular senescence in the lungs and emphysema at a young age [103]. While no specific therapy exists yet for telomerase-mutant COPD aside from lung transplantation, identifying this endotype has implications for family counseling and overlaps with research into anti-aging therapeutics. These genetic endotypes are rare but highlight how distinct the underlying mechanism can be (proteinase-antiprotease imbalance in AAT vs. defective cell renewal in telomerase mutation).

### Persistent inflammation/autoimmune endotype

A subset of COPD patients exhibit evidence of systemic autoimmune features or persistent inflammation unlinked to smoking exposure alone [104–106]. For instance, some have high titers of autoantibodies (against epithelial or extracellular matrix components) and lung histopathology showing lymphoid follicles [107], resembling an autoimmune process [108, 109]. This endotype is still being characterized, but it may explain severe disease in some former smokers or never-smokers [19]. Therapies targeting autoimmune pathways (e.g. B-cell depletion or cytokine inhibition) are speculative at this stage, but clinical trials are considering anti-inflammatory strategies beyond corticosteroids for such patients.

### Pulmonary vascular endotype

A minority of COPD patients develop disproportionate pulmonary hypertension relative to their airway obstruction. These patients exhibit a pulmonary vascular phenotype marked by severe dyspnea and very low diffusing capacity out of proportion to spirometric impairment, due to extensive vascular remodeling in the lungs [110]. Recent work implicates endothelial hypoxia-inducible factor (HIF)−2α (EPAS1) signaling as a mechanistic axis that pushes disease toward a vascular-dominant endotype. In healthy individuals, HIF-2α activity is maintained at a low but steady basal level, sufficient to support vascular endothelial growth factor (VEGF) signaling, endothelial survival, and alveolar–capillary maintenance, while avoiding excessive vascular remodeling. This homeostatic balance ensures the integrity of both vascular and parenchymal compartments.

When this equilibrium is disturbed, divergent pathological trajectories emerge. Sustained HIF-2α activation (e.g., due to chronic hypoxia or PHD2 [prolyl hydroxylase domain protein 2] loss) stabilizes endothelial HIF-2α, which (i) induces endothelial-to-mesenchymal transition (EndoMT) via SNAI1/2 [111], (ii) up-regulates vasoconstrictor and remodeling programs such as endothelin-1 and a HIF-2α–arginase pathway that reduces nitric oxide (NO) bioavailability [112], and (iii) releases paracrine cues that drive distal arterial muscularization and obliterative remodeling. Collectively, these processes produce pulmonary hypertension and define the vascular-dominant endotype of COPD [113]. 

Conversely, loss of endothelial HIF-2α disrupts the alveolar–capillary niche and precipitates emphysema: endothelial Hif-2α deletion or VEGF-pathway blockade in mice causes airspace enlargement, whereas restoring VEGF preserves alveolarization [114]. This bidirectional effect, with overactivation driving vascular remodeling and deficiency leading to emphysematous destruction, supports HIF-2α as a molecular “switch” between vascular-dominant and airway/emphysema-dominant trajectories in COPD [110]. 

Although pulmonary vasodilator therapies are not standard for COPD-PH outside trials, preclinical HIF-2α inhibition (e.g., PT2385, PT2567; genetic reversal of HIF-2–driven remodeling) can attenuate established pulmonary hypertension and reverse vascular changes while identifying the HIF-2α axis as a tractable target in patients with a vascular endotype, awaiting biomarker-guided enrichment and clinical validation [115]. 

The increasingly sophisticated omics technologies are unveiling COPD endotypes that cannot be identified through clinical observation alone. Through integrated analyses of genomics, transcriptomics, proteomics, and metabolomics, researchers have revealed distinct molecular signatures in COPD patients. For instance, a recent multi-layered network analysis of lung tissue integrating mRNA, microRNA, and DNA methylation data revealed five molecular patient clusters with divergent underlying biological characteristics [116]. Remarkably, some of these molecular subgroups had similar clinical features yet different endotypes: for example, two groups both had severe airflow limitation and emphysema, but one showed high T-cell/B-cell immune signatures with loss of club cells, whereas the other did not [116]. This indicates that distinct endotypes can underlie the same phenotype of severe emphysema, which may explain why responses to therapy vary even among patients who look clinically similar. Such findings underscore the importance of moving beyond surface phenotypes to the molecular “fingerprint” of each patient’s disease.

COPD endotypes are not isolated compartments – they interact and often coexist. For example, chronic colonization with bacteria (“infection endotype”) will drive neutrophilic inflammation; conversely, long-term ICS treatment for eosinophilic COPD can increase pneumonia risk and potentially shift microbiome balance [117, 118]. Likewise, an individual with eosinophilic inflammation may also suffer from excessive mucus production. This interaction means that targeting one pathway might not fully address the disease. The inconsistent clinical efficacy of single-cytokine inhibitors (e.g. anti–IL-5 or anti–IL-17 alone) in COPD likely owes to this network effect [117], where multiple inflammatory loops compensate or parallel one another, blunting the impact of pathway-specific inhibition. Nonetheless, recent positive results with IL-5 and IL-4Rα blockade in biomarker-enriched subgroups suggest that efficacy can be achieved when treatment is mechanism-matched and patients are appropriately stratified [96, 99]. Therefore, precision medicine approaches increasingly recognize that combinatorial therapies or broad-modulating strategies (like upstream “alarmin” cytokine blockers, or even non-immune modulators like senolytics for aging/senescence pathways) might be required to significantly alter disease trajectories. Ongoing research is working to refine endotype definitions and identify dominant drivers for each patient, which could become therapeutic targets [117]. 

## Candidate biomarkers of COPD

Identifying phenotypes and endotypes in practice relies on biomarkers—measurable indicators of specific disease processes. In COPD, biomarkers span a spectrum ranging from clinical surrogates (e.g., chronic sputum production as a marker of mucus hypersecretion) to sophisticated molecular assays. An ideal biomarker in COPD may function in one or more of the following capacities: predicting prognosis (e.g., risk of exacerbations or mortality), predicting or monitoring therapeutic responses (i.e., theragnostic biomarkers), or elucidating underlying endotypes to enable targeted therapies (endotype classifiers). Below, we examine key established and emerging biomarkers in COPD, emphasizing their roles in prognostication, treatment personalization, and mechanistic characterization of disease heterogeneity.

### Circulating biomarkers

The peripheral eosinophil count has emerged as one of the most useful biomarkers in COPD [119, 120]. It is readily available and reflects type 2-high airway inflammation [94]. Blood eosinophils correlate with tissue eosinophil infiltration (albeit imperfectly) [118], and numerous studies have shown they predict the benefit from ICS therapy [118]. Patients with higher eosinophil counts have more frequent exacerbations if not on ICS [80], and derive greater reduction in exacerbations when ICS is added to therapy [121]. GOLD guidelines now use blood eosinophils to guide therapy: >300 cells/µL indicates a high likelihood of benefit from ICS (exacerbation reduction), while < 100/µL suggests little benefit [118]. Importantly, other studies have employed alternative thresholds, such as ≥ 150 cells/µL, to identify patients likely to respond, reflecting variability across different cohorts [122]. This reinforces the view that blood eosinophil counts should be interpreted as a continuous risk gradient rather than rigid cut-offs, with higher counts generally predicting greater ICS benefit [118]. Clinically, a blood eosinophil count measured during stability can identify eosinophilic endotype patients, thereby guiding the use of ICS or even biologics. It’s worth noting eosinophil counts can fluctuate due to infections or steroids [117], so repeat measurements may be useful.

In addition to eosinophils, various blood proteins offer a minimally invasive strategy for investigating systemic inflammation, oxidative stress, and molecular signatures associated with COPD pathogenesis [123], while concurrently carrying prognostic significance. Acute-phase reactants have been investigated as biomarkers linked to exacerbations: C-reactive protein (CRP) demonstrates association with COPD exacerbations. Though nonspecific, persistent elevation in COPD patients indicates heightened risks of exacerbations and comorbidities. In a randomized trial, antibiotic stewardship protocols guided by point-of-care CRP testing during COPD exacerbations reduced antibiotic usage without evidence of harm [124]. Nevertheless, as a non-specific inflammatory marker, CRP can also be elevated in conditions such as acute infections or cardiovascular disease, and therefore its predictive value when used alone should be interpreted cautiously in the appropriate clinical context.

Elevated plasma fibrinogen and soluble receptor for advanced glycation end products (sRAGE) levels in numerous COPD patients with systemic inflammation correlate with reduced pulmonary function, COPD symptoms, and exacerbation frequency, enabling risk stratification of high-risk populations prone to frequent exacerbations [125–127]. Currently, fibrinogen is also employed as a biomarker to enrich clinical trial cohorts (e.g., exclusively enrolling patients with elevated fibrinogen to amplify adverse event rates). Other plasma biomarkers under investigation include surfactant protein D (SP-D) and Clara cell protein 16 (CC16)—both pulmonary-derived proteins whose diminished levels reflect airway epithelial injury (reduced CC16 associates with rapid lung function decline and exacerbations) [128]. 

IL-6 and IL-8 profiles exhibit distinct clinical implications: elevated IL-6 correlates with mortality and muscle wasting, while IL-8 reflects neutrophilic inflammation [129]. Composite inflammatory scores integrating CRP, IL-6, and IL-8 may provide superior risk stratification compared to individual biomarkers [130, 131]. Oxidative stress biomarkers such as malondialdehyde (MDA) and oxidized DNA/RNA products elucidate systemic redox imbalance observed in COPD [132]. 

Circulating biomarkers indicative of extracellular matrix remodeling (e.g., MMPs, tissue inhibitors of metalloproteinases [TIMPs]) and endothelial dysfunction (e.g., endothelin-1, von Willebrand factor) may offer prognostic insights into disease progression and cardiovascular complications in COPD patients [133]. Finally, genetic markers (e.g., specific gene polymorphisms) and epigenetic signatures (e.g., DNA methylation patterns) are being explored as stable biomarkers for susceptibility and phenotypic characterization, though currently remaining investigational tools.

### Sputum biomarkers

Analysis of induced sputum provides a window into airway inflammation [134]. The cellular composition of sputum, particularly the eosinophil-to-neutrophil ratio, defines inflammatory phenotypes (in research settings, a threshold of ≥ 3% sputum eosinophils typically delineates eosinophilic COPD). Sputum eosinophilia demonstrates predictive value for responsiveness to corticosteroid therapy; however, sputum acquisition remains more technically challenging than measuring blood eosinophil counts. Cytokine profiling in sputum reveals elevated levels of IL-8, IL-1β, and TNF in neutrophilic COPD [135], while eosinophil cationic protein (ECP) and periostin (an IL-13-induced protein) have been investigated in eosinophilic variants. Gene expression and microRNA profiling of sputum are emerging research tools to identify endotypes; for example, a high IL-17 gene signature could identify the Th17 endotype. Neutrophil elastase (NE), a serine protease secreted by neutrophils, shows increased concentrations in sputum during bacterial infections among COPD patients [135]. Furthermore, sputum proteomic and metabolomic analyses demonstrate potential for identifying novel molecular signatures associated with COPD phenotypes and therapeutic responses.

### Lung tissue-based biomarkers

Lung tissue-derived biomarkers have attracted considerable attention due to their unique capacity to directly reflect pathological characteristics at disease sites [136]. Compared to peripheral biomarkers in serum or sputum, pulmonary tissue specimens offer enhanced precision in delineating airway remodeling, alveolar destruction, and molecular dynamics within local microenvironments. For instance, elevated activity of MMPs in lung parenchyma, accumulation of elastic fiber degradation products, or altered infiltration ratios of specific immune cells (e.g., CD8^+^ T lymphocytes and M1 macrophages) demonstrate direct correlations with pathological progression of small airway fibrosis or emphysema. These tissue-specific markers circumvent confounding factors inherent to systemic inflammatory mediators in serum (e.g., CRP, IL-6) while overcoming biases in sputum samples caused by upper respiratory contamination or cellular viability variations. Their unique value is particularly evident when evaluating core pathogenic mechanisms such as protease-antiprotease imbalance and oxidative stress pathway activation. However, clinical implementation remains constrained by risks associated with invasive biopsies, with current research primarily utilizing surgically resected specimens or autopsy materials. This limitation has spurred innovation in emerging technologies including imaging-guided minimally invasive sampling and single-cell spatial transcriptomic analysis as potential solutions to overcome current methodological barriers.

Lung tissue samples obtained from former smokers undergoing clinically indicated thoracic surgeries have been instrumental in multi-omics investigations of COPD pathogenesis. In one analytical framework, researchers identified 4,997 genes associated with expression quantitative trait loci (eQTLs) below genome-wide significance thresholds, subsequently constructing gene regulatory networks. The top-ranked gene, *MAPT*, had been previously identified in an independent GWAS [137]. A follow-up study employing network-based clustering methodologies integrated gene expression and DNA methylation profiles, revealing that clusters enriched with COPD subjects demonstrated overrepresentation of transcripts involved in IL signaling and immune regulatory interactions between lymphoid and non-lymphoid cells [138]. Mass spectrometry-based proteomic profiling of 100 COPD patients and 52 controls identified 25 protein biomarkers significantly associated with COPD, including AGRN, ANXA2, and GPRC5A [139]. Single-cell RNA sequencing of lung tissues from 17 COPD patients versus 15 age-matched controls revealed diminished stress tolerance in a HHIP-expressing alveolar type 2 cell subpopulation among COPD subjects [140]. Collectively, these findings exemplify how pulmonary multi-omics approaches effectively bridge COPD genetic risk markers with targeted molecular pathways underlying disease pathology.

### Biomarkers based on type 2/non-type 2 inflammatory microenvironments

Early observations indicated the involvement of neutrophils, CD8^+^ Tc1 cells, CD4^+^ Th1/Th17 cells, macrophages, and natural killer (NK) cells in COPD airway inflammation [69, 141]. Subsequent studies revealed that a subgroup of patients with exacerbations may also exhibit heightened eosinophilic inflammation [142]. These patients, identified as having ACO [143], demonstrate elevated Th2 cell signatures and exhibit favorable responses to ICS [144]. The inflammatory microenvironment within the airway epithelium induces inducible NO synthase (iNOS), leading to NO release [145]. FeNO serves as another biomarker for eosinophilic airway inflammation in COPD [146]. Elevated FeNO levels during hospitalization in COPD exacerbation patients may predict therapeutic responsiveness, showing positive correlations with improvements in FEV_1_ and negative associations with hospitalization duration [147]. Exhaled breath condensate (EBC) from COPD patients contains LTB4 (a potent neutrophil chemoattractant) at concentrations 2.5-fold higher than healthy controls and prostaglandin E2 (PGE2) levels 2.2-fold elevated [148]. Bronchial epithelial cells, as the primary anatomical barrier exposed to harmful cigarette smoke particles, initiate airway remodeling through the release of diverse proinflammatory mediators [149]. Epidermal growth factor receptor (EGFR) signaling is a key regulator of epithelial function and proliferation. Smoking induces a distal-to-proximal reorientation of small airway epithelium [150], enhancing EGFR signaling and exacerbating COPD phenotypes such as goblet cell hyperplasia, mucus hypersecretion, and epithelial-mesenchymal transition (EMT) [151]. Furthermore, nicotine in cigarette smoke activates the Wnt3a signaling pathway, promoting β-catenin nuclear translocation and inducing canonical EMT features [152, 153]. Multiple EMT markers have been reported, including α-smooth muscle actin (α-SMA), vimentin, fibroblast-specific protein-1 (FSP-1/S100A4), desmin, (pro)-collagen, fibronectin, CTGF, N-cadherin, the Snail, Slug, Twist, β-catenin-bounded transcription factors, and MMPs [154, 155]. COPD patients also exhibit elevated hydrogen peroxide and lipid peroxidation products in EBC, reflecting smoking-induced oxidative stress [156]. Researchers are exploring “breathomics”—the analysis of exhaled volatile organic compounds (VOCs)—as non-invasive signatures for COPD endotypes (e.g., specific VOC patterns may indicate bacterial colonization or eosinophilic inflammation) [157, 158]. While promising, these technologies remain investigational and are not yet ready for routine clinical application.

### Imaging biomarkers

The imaging biomarkers of COPD are primarily manifested as structural alterations in the pulmonary parenchyma, airways, and vascular system [159]. High-resolution CT (HRCT) serves as the primary modality for evaluating COPD, with characteristic findings encompassing emphysema, airway remodeling, and pulmonary vascular abnormalities. On CT, emphysema manifests as focal or diffuse areas of low attenuation without visible walls, predominantly involving the upper lobes. It can be classified into centrilobular, panlobular, and paraseptal subtypes. The CT emphysema index, defined as the percentage of lung voxels below − 950 Hounsfield units (HU), correlates with physiological decline and mortality risk. Bronchial wall thickening and lumen narrowing reflect chronic inflammatory airway remodeling. Parameters such as pi10 (calculated from the square root of the airway wall area for a hypothetical 10 mm diameter airway) quantify airway remodeling to identify phenotypes dominated by bronchial pathology [160]. 

Mucus plugging on chest CT is common in COPD (≈ 25–67%), often persists over time, and is associated with lower FEV_1_, worse quality of life, and higher risks of exacerbations and mortality [161, 162]. Recent cohort analyses show that involvement of multiple bronchial segments by mucus plugs confers a stepwise increase in risk and can act as an imaging marker of high-risk disease [163]. These observations support systematic scoring of mucus plugs in quantitative CT workflows and consideration of mucus-targeted interventions in selected patients.

Vascular abnormalities manifest as peripheral vascular pruning and pulmonary hypertension-associated main pulmonary artery dilation. Air trapping on expiratory CT appears as mosaic attenuation, indicating small airway dysfunction. New imaging tools like PRM enhance diagnostic granularity by integrating inspiratory and expiratory CT data to spatially differentiate emphysematous tissue (PRM_emph_) from functional small-airway disease manifesting as air trapping (PRM_fSAD_) [159], thereby distinguishing patients whose airflow limitation stems predominantly from alveolar destruction versus small-airway dysfunction [164]. 

Functional imaging modalities complement these structural evaluations: hyperpolarized gas magnetic resonance imaging (MRI) maps regional ventilation patterns with sensitivity to detect small-airway abnormalities prior to observable CT changes, while positron emission tomography (PET)-MRI fusion imaging offers insights into localized inflammation and perfusion defects that could guide targeted anti-inflammatory therapies.

The clinical translation of these biomarkers is reshaping therapeutic decision-making. CT-defined emphysema distribution patterns directly inform eligibility for lung volume reduction surgery, particularly in upper-lobe predominant disease. Similarly, detecting covert bronchiectasis via CT in chronic bronchitis patients necessitates adjunct therapies like airway clearance maneuvers and prophylactic antibiotics. PRM-based endotyping holds promise for personalized interventions, as patients with high PRM_emph_ burden may derive optimal benefit from volume-reduction strategies.

Emerging technologies like radiomics and AI-driven image analysis revolutionize COPD subtyping through automated extraction of imperceptible features—textural heterogeneity and vascular morphometry [165]. These innovations emphasize a paradigm shift toward precision medicine, where imaging-guided management increasingly replaces symptom-driven approaches. Continuous technological advancements will further refine diagnostic accuracy and therapeutic personalization in COPD care [166]. 

## Treatable traits approach

While phenotypes and endotypes classify COPD at a population level, the treatable traits strategy operationalizes precision medicine at the individual patient level [167]. Treatable traits are defined as specific clinical or biological characteristics that can be identified in a patient and treated independently to improve outcomes [168]. This approach acknowledges that a given patient may have several coexisting traits contributing to their health status, spanning pulmonary aspects, extrapulmonary features, and behavioral/risk factors [169]. Instead of focusing on the umbrella label “COPD,” clinicians systematically assess for a roster of treatable problems and address each with targeted therapy.

The treatable traits paradigm was first extensively applied in severe asthma and is now being adapted for COPD [170]. This approach involves two key steps: first, conducting a comprehensive multidimensional assessment to identify all clinically relevant traits, and second, implementing targeted evidence-based interventions for each identified trait. These traits are systematically categorized into three primary domains [171]. 

Pulmonary traits encompass specific lung-related pathological features. Airway eosinophilia may be managed with ICS or anti-IL5 biologics, while chronic bronchitis with mucus hypersecretion often requires mucolytics combined with airway clearance techniques. Patients experiencing frequent exacerbations benefit from prophylactic therapies, and those with fixed airflow obstruction accompanied by hyperinflation typically require a combination of bronchodilators, pulmonary rehabilitation, and potentially lung volume reduction procedures. Comorbid conditions like bronchiectasis warrant antibiotic therapy and physiotherapy, whereas ACO should follow asthma management protocols. Notably, upper airway pathologies such as chronic rhinosinusitis represent treatable traits whose management can positively influence lower airway symptoms.

Extrapulmonary traits address systemic manifestations and comorbidities. Nutritional interventions and exercise programs form the cornerstone for managing sarcopenia or cachexia, sometimes supplemented with anabolic agents. Cardiovascular risk reduction involves aggressive risk factor modification and judicious use of β-blockers. Osteoporosis management includes calcium/vitamin D supplementation and bisphosphonates, while psychological comorbidities like anxiety/depression require integrated approaches combining behavioral therapy and pulmonary rehabilitation. Persistent systemic inflammation, though lacking direct therapeutic targets, serves as a biomarker for heightened monitoring and potential eligibility for anti-inflammatory clinical trials.

Behavioral/risk factor traits focus on modifiable health behaviors and environmental exposures. Smoking cessation support remains paramount, complemented by strategies to improve inhaler technique and medication adherence through patient education and regimen simplification. Physical inactivity requires structured pulmonary rehabilitation programs, while environmental exposures demand protective measures against pollutants and occupational hazards. Socioeconomic factors including health literacy deficits and social isolation necessitate tailored interventions through support networks and educational initiatives. Addressing these traits not only slows disease progression but also potentiates the effectiveness of other targeted therapies.

The GOLD guidelines recognize dyspnea and the occurrence of exacerbations as two pivotal identifiable and treatable traits in COPD [18, 172]. Both the COPD assessment Test (CAT) score and modified Medical Research Council (mMRC) scale serve as valuable biomarkers for quantifying breathlessness severity and informing therapeutic strategies. Based on these assessment tools, COPD-related dyspnea can be stratified into mild and severe symptom categories. Therapeutic objectives focus on alleviating respiratory distress, enhancing pulmonary function, improving exercise tolerance, and ultimately optimizing quality of life, with lung volume reduction surgery emerging as a potential consideration in selected cases. Acute exacerbations, characterized by abrupt symptom deterioration, present substantial clinical challenges in COPD management, with intervention strategies encompassing vaccination protocols, corticosteroid/bronchodilator (ICS/LABA/LAMA) regimens, and patient education for early symptom recognition [173]. 

Emerging evidence highlights chronic bronchial bacterial colonization as another critical treatable trait requiring targeted intervention [174]. Contemporary research reveals significant correlations between airway microbiota composition and various clinical parameters including COPD phenotypic characteristics, disease severity, and long-term mortality [175]. The respiratory microbiome represents a promising therapeutic target, serving both as a potential biomarker for patient stratification and a modifiable element in precision medicine paradigms [176]. This microbial ecosystem demonstrates clinical malleability through therapeutic interventions, offering untapped opportunities for tailored treatment approaches [176]. However, distinct microbial patterns observed in neutrophilic versus eosinophilic COPD endotypes necessitate differentiated therapeutic strategies, reflecting the pathobiological heterogeneity inherent in COPD manifestations [177]. 

The treatable traits approach, fundamentally aligned with precision medicine paradigms, acknowledges the multidimensional nature of individual patients as composite entities comprising multiple characteristics that exist along dynamic spectrums of expression [178], rather than constraining them within rigid phenotypic classifications. This “label-free” methodology eschews traditional disease categorization in favor of directly targeting the constituent elements of a patient’s clinical profile [179]. By treating a patient’s unique combination of traits, this approach aims to maximize benefit (e.g. improved quality of life, reduced exacerbations) while avoiding unnecessary treatments. For example, a patient without eosinophilic inflammation (a trait absent) would be spared high-dose ICS (thus avoiding side-effects), whereas a patient with that trait gets the ICS they need.

Increasing amounts of empirical evidence substantiates the clinical validity of this model. Implementation of multidimensional trait-based assessments and management in COPD populations has demonstrated superior improvements in health status compared to conventional care [168], with sustained benefits observed at both 6- and 12-month follow-up intervals [168]. Parallel applications in severe asthma management have shown analogous enhancements in symptom control and quality metrics, underscoring the transdiagnostic applicability of this framework across chronic respiratory conditions [180]. While large-scale randomized trials remain necessary to confirm COPD-specific outcomes, current data suggest trait-targeted interventions achieve therapeutic gains beyond standard care parameters [181]. 

Major clinical guidelines increasingly endorse this paradigm, with both GOLD and The Lancet Commission advocating enhanced phenotypic and biological characterization to inform personalized COPD management [3, 7]. Practical implementation challenges persist, however, particularly regarding the temporal and resource demands of comprehensive trait profiling, which typically necessitates multidisciplinary expertise (e.g., psychological or nutritional specialists) [168]. Resource-constrained settings may require strategic prioritization of high-impact traits, with contextual adaptation proving essential [168]. In low-income regions, targeting modifiable risk factors like indoor air pollution and smoking cessation might yield maximal benefit, whereas high-resource environments could concurrently address nuanced biological traits alongside advanced therapies [182]. Health system evolution will be critical to support this transition, potentially involving specialist referral networks for complex traits, provider education programs, and integration of decision-support tools for trait identification [182]. 

Ultimately, the treatable traits paradigm transcends the traditional one-size-fits-all approach through personalized therapeutic targeting strategies, enabling the implementation of precision medicine for COPD. Conceptually distinct from phenotypic and endotypic systems that provide population-based research frameworks, it nevertheless draws upon both when identifying specific characteristics in individual patients. Proven clinical advantages and increasing incorporation into guidelines signal an impending paradigm shift towards personalized respiratory care; however, systematic adaptation remains essential to fully realize its potential across diverse healthcare settings [168]. 

## Conclusions and future directions

COPD has entered the era of precision medicine, driven by evolving insights into its heterogeneous phenotypes, distinct endotypes, and treatable traits. Contemporary understanding recognizes COPD not as a singular entity but as a convergence of interrelated biological pathways manifesting as airflow limitation [183]. By classifying patients into clinically meaningful phenotypes (e.g., chronic bronchitis–, emphysema-predominant, exacerbation-prone) and investigating dominant inflammatory endotypes (eosinophilic, neutrophilic, etc.), clinicians can tailor therapeutic strategies with enhanced individual specificity [184]. Concretely, blood eosinophils are now embedded in guideline-directed therapy to estimate the likelihood of benefit from ICS (greatest at ≥ 300 cells/µL; minimal at < 100 cells/µL), operationalizing a continuous, biomarker-based approach in routine care. Quantitative imaging, particularly PRM, can spatially distinguish functional small-airway disease from emphysema and map their trajectories, an insight that refines endotyping and informs intervention selection [185, 186]. Moreover, phase-3 trials in biomarker-enriched COPD populations have shown reduced exacerbations with dupilumab (anti-IL-4Rα) and mepolizumab (anti-IL-5) added to optimized inhaled therapy, marking tangible progress for the eosinophil/type-2–high endotype.

The coming years will likely see combinatorial biomarker algorithms that integrate readily available clinical metrics (e.g., blood eosinophils), cytokine profiles, and quantitative imaging parameters (e.g., PRM-defined fSAD/emphysema) to guide therapeutic allocation and clinical-trial enrichment. Machine-learning applications to multidimensional datasets are enabling data-driven subgroups with potential therapeutic implications, but translation will require harmonized pipelines and transparent model reporting [44]. At the same time, technical bottlenecks remain: multi-omics integration is challenged by batch effects, cross-platform normalization, and limited external validation, while sample-size and reproducibility constraints can inflate effect estimates. Importantly, key biomarkers show temporal variability even during clinical stability, underscoring the need for repeat measurements and longitudinal anchoring of endotypes [187]. 

On the therapeutic front, while no single “magic bullet” has emerged, the pipeline is rich. Biologics targeting upstream alarmins (e.g., IL-33/TSLP) and downstream cytokines (e.g., IL-1β, IL-6) aim to disrupt inflammatory redundancy; cell-based and regenerative approaches represent long-term precision strategies for emphysema. Precision principles are also permeating rehabilitation, identifying non-responders who may benefit from adjunct anabolic strategies or tailored exercise regimens, and pharmacogenomic signals continue to be explored for bronchodilator response. Implementation, however, must balance individualization with public-health fundamentals: smoking cessation and equitable access to guideline-based care remain central. Economic considerations are non-trivial, with cost-effectiveness often driven by baseline exacerbation risk and mortality effects, and varying across patient subgroups, which highlights the importance of responder enrichment in both trials and practice [188]. Encouragingly, many precision tools (e.g., eosinophil counts) are low-cost, and recently phase-3 programs MATINEE (mepolizumab) [96] and BOREAS/NOTUS (dupilumab) [189] demonstrate outcome gains in biomarker-defined COPD.

This evolving paradigm is akin to oncology’s mutation-driven treatment models, positioning COPD management at the threshold of personalized care. Through comprehensive characterization of individual phenotypic signatures, inflammatory endotypes, and treatable traits, clinicians can implement multidimensional management strategies. Priorities now include standardized, externally validated multi-omic pipelines; longitudinal, outcome-anchored endotyping to capture dynamic biology; pragmatic trial designs that test biomarker-guided strategies; and robust health-economic evaluations of treatable-trait programs across diverse health systems. If achieved, these steps should translate into fewer exacerbations, better quality of life, slower disease progression, and a shift from terminal trajectories to chronic, controllable care tailored to the individual. As evidence accumulates, precision medicine approaches may fulfill their potential to rewrite the therapeutic narrative of this complex respiratory syndrome.

## Data Availability

No datasets were generated or analysed during the current study.

## Abbreviations

AAT: α_1_-antitrypsin
ACO: Asthma-COPD overlap
AECOPD: Acute exacerbation of COPD
α-SMA: α-smooth muscle actin
CAT: COPD assessment Test
CC16: Clara cell protein 16
COPD: Chronic obstructive pulmonary disease
CRP: C-reactive protein
CT: Computed tomography
CTGF: Connective tissue growth factor
DAMP: Damage-associated molecular pattern
DC: Dendritic cell
DL_CO_: Diffusing capacity for carbon monoxide
EBC: Exhaled breath condensate
ECP: Eosinophil cationic protein
EGFR: Epidermal growth factor receptor
EMT: Epithelial-mesenchymal transition
eQTL: Eexpression quantitative trait loci
FeNO: Fractional exhaled nitric oxide
FEV_1_: Forced expiratory volume in one second
FSP-1: Fibroblast-specific protein-1
FVC: Forced vital capacity
GOLD: Global Initiative for Chronic Obstructive Lung Disease
HIF: Hypoxia-inducible factor
ICS: Inhaled corticosteroids
IL: Interleukin
ILA: Interstitial lung abnormality
ILC: Innate lymphoid cell
ILD: Interstitial lung disease
iNOS: Inducible nitric oxide synthase
IPF: Idiopathic pulmonary fibrosis
LABA: Long-acting β-agonist
LAMA: Long-acting muscarinic antagonist
LT: Leukotriene
MAPK: Mitogen-activated protein kinase
MDA: Malondialdehyde
MMP: Matrix metalloproteinase
mMRC: Modified Medical Research Council
NE: Neutrophil elastase
NF-κB: Nuclear factor-κB
NK cell: Natural killer cell
NO: Nitric oxide
OR: Odds ratios
PAMP: Pathogen-associated molecular pattern
PDE4: Phosphodiesterase-4
PG: Prostaglandin
PRISm: Preserved Ratio Impaired Spirometry
PRM: Parametric response mapping
SP-D: Surfactant protein D
sRAGE: Soluble receptor for advanced glycation end products
Tc cell: Cytotoxic T cell
Th cell: T helper cell
TIMP: Tissue inhibitors of metalloproteinase
TSLP: Thymic stromal lymphopoietin
VEGF: Vascular endothelial growth factor
VOCs: Volatile organic compounds
